# Human African trypanosomiasis (HAT) in the Republic of Congo: why the Congolese population is reluctant to screening?

**DOI:** 10.11604/pamj.2022.42.309.34830

**Published:** 2022-08-25

**Authors:** Viny Andzi Elenga, Abel Lissom, Christevy Vouvoungui, Tsengue-Tsengue, Gabriel Ahombo, Francine Ntoumi

**Affiliations:** 1 Fondation Congolaise Pour la Recherche Médicale, Brazzaville, République du Congo,; 2 Faculté des Sciences et Techniques, Université Marien Ngouabi, Brazzaville, République du Congo,; 3 Department of Biological Science, Faculty of Science, University of Bamenda, Bamenda, Cameroon,; 4 Institute of Tropical Medicine, University of Tübingen, Germany

**Keywords:** Human African trypanosomiasis, screening, knowledge, perception, Republic of Congo

## Abstract

**Introduction:**

human African trypanosomiasis (HAT) is a neglected tropical infection, and surveillance of the disease relies on community participation in screening. This study aimed to identify the main factors associated with low community uptake of the HAT screening in endemic districts in the Republic of Congo.

**Methods:**

a cross-sectional survey was carried out during a sensitisation campaign about HAT in the districts of Mpouya, Ngabé and Loudima, which are endemic for the disease. After signing the informed consent form, participants were organized into groups of 10 for focus group discussions (FGDs). A list of questions was used for guiding the discussion, addressing understanding of the disease and reasons for refusing screening.

**Results:**

out of 220 recruited individuals (corresponding to 22 FGDs), 58.6% were men. The majority of the respondents described HAT as a rural disease (48.2%) or as a witchcraft (22.3%). Among the clinical signs cited by the participants, sleep disorder (40%) was the most common answer, followed by prolonged fever (19.5%) and madness (14.1%). The main reasons for non-adherence to HAT screening was the fear of lumbar puncture (45.9%) and stigmatisation (22.3%).

**Conclusion:**

the findings of this study suggest that more effort should be put into raising awareness of HAT and the benefits of screening amongst the Congolese population, in order to strengthen the national disease control program.

## Introduction

Human African trypanosomiasis (HAT) is classified as one of the world´s classical “neglected tropical diseases” that is endemic and represents a major threat to public health in 36 sub-Saharan Africa countries [1]. It is caused by two parasite species of Trypanosoma namely: *Trypanosoma brucei gambiense* (*T.b. gambiense*) and *Trypanosoma brucei rhodesiense* (*T.b. rhodesiense*). *T.b. gambiense* currently accounts for 95% of reported cases of sleeping sickness and causes a chronic infection in West and Central Africa [2]. *Trypanosoma brucei rhodesiense* (*T.b. rhodesiense*) is the pathogenic agent for the more acute form of the disease, which is endemic in Eastern Africa [3,4].

The epidemiology of the HAT is mediated by the interaction of the parasite (trypanosome) with the vectors (tsetse flies), as well as with the human and animal hosts within a particular environment. Related to these interactions, the disease is confined in spatially limited areas called “foci” within sub-Saharan Africa, mainly in remote rural areas. There has been a significant decline in new cases of HAT over the past 20 years, because of massive and concerted collaborative efforts to control the disease by World Health Organization (WHO), non-government organizations, African governments, the pharmaceutical industry, and research charities and institutions. Key to this disease reduction were the introduction of rapid detection, isolation of patients, and treatment of new HAT cases to prevent disease spread and also to provide more effective vector control.

The goal of WHO was to eliminate sleeping sickness as a public health problem by 2020 and to achieve interruption of its transmission by 2030 [5]. These goals could be achievable, though there are several challenges and caveats that should be taken into account. The fact that the disease is mainly confined to foci located in remote rural areas poses a serious problem for effective screening of HAT, which is likely to result in the underestimation of infection. In addition, the socio-cultural behaviour may be a major challenge for engaging the community in this effort. In Central Africa, while the Democratic Republic of the Congo reported 70% of cases, the Republic of Congo (RoC) declared between 10 and 100 new cases in 2019 [6]. The RoC is considered as an endemic country for HAT, and there is a need for updated epidemiological data, especially in remote areas at risk. Thus, understanding the factors associated with adherence of the local population to the HAT infection screening in villages where disease is known to be present is a key component of the control and elimination of the disease. The present study aimed to assess the socio-cultural factors associated with the adherence of the Congolese population to the HAT screening in three endemic districts of RoC.

## Methods

**Figure 1:**
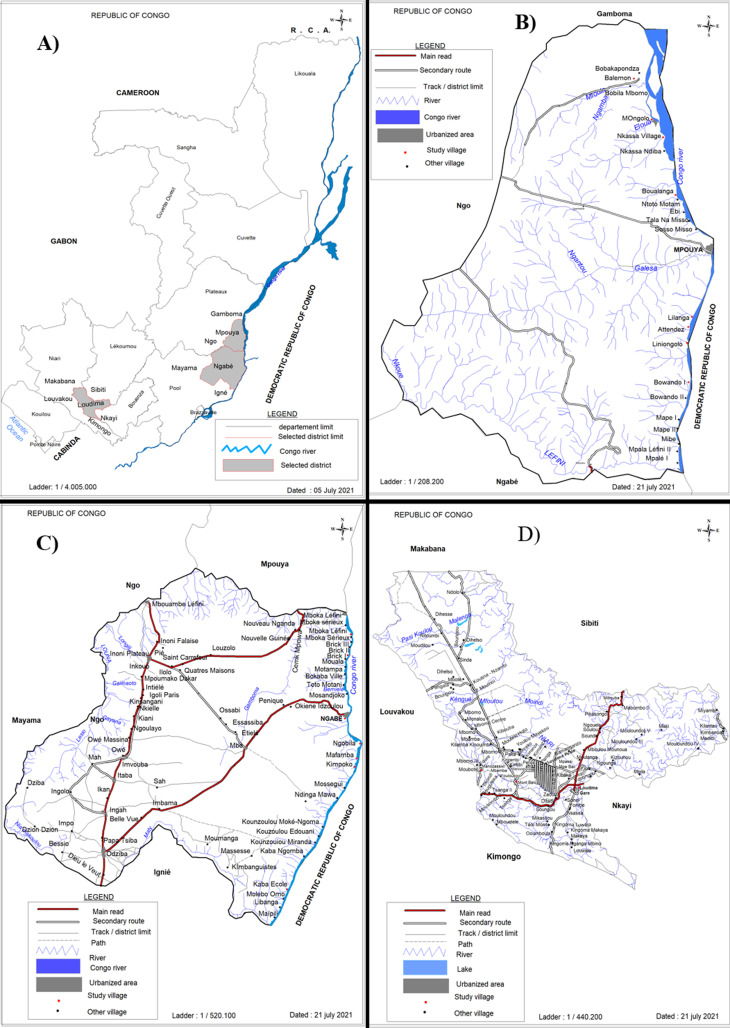
map of the foci at risk of the human African trypanosomiasis selected for the study; A) the map of Republic of Congo bringing out the three districts of the study; B) map of the district of Mpouya with the study villages; C) map of the district of Ngabe with the study villages; D) map of the district of Loudima with the study villages

**Study areas:** this was a cross-sectional study carried out in the endemic foci of the RoC (Figure 1A) located in the districts of Mpouya (Figure 1B) in plateaux department, Ngabé (Figure 1C) in pool department and Loudima (Figure 1D) located in the department of Bouenza (Figure 1). These districts have a predominantly tropical savanna climate. Mpouya is a region located at 2°36’57” South, 16°12´43´´ East with an altitude of 305 m. It is located near the Congo River and is approximately 215 km from Brazzaville, the Congo's capital city. There are around 9,000 inhabitants, who are mainly involved in agriculture and fishing activities. Loudima is geo-localized at 4°06´09´´ South, 13°02´58´´ East. It has around 18,000 inhabitants and agriculture is the main economic activity of the region. The city of Ngabé, localised at 3°12’ 52’’ South, 16° 10’ 1’’ East with an altitude of 294 m, has 30,000 inhabitants.

**Study design and data collection:** during a sensitisation campaign organized by the National Control Program for Human African Trypanosomiasis and the Organization of Coordination for the Fight against Endemic Diseases in Central Africa (OCEAC) a cross-sectional descriptive survey was conducted. The unit of analysis was the individual participant. This study was conducted between June and September 2019. According to the sample size calculator Raosoft 2004, a sample size of 195 respondents was estimated, assuming that the total size of population of the study areas was 57,000, a confidence level was fixed at 95%, an error margin of 7% and a response distribution of 50%. The below formula was used to determine the sample size:


$$n=\frac{Nx}{\left((N-1)E^{2}+x\right)}$$

Where n is the sample size; E is the marginal error; N is the population size of the locality; and x is the confidence level. The selection criterion for enrolment were: 1) to be aged over 18 years; 2) to practice fishing, hunting or farming; 3) to be permanent residents in one of the 3 endemic foci for at least the last 2 years. The study was carried out following administrative procedure required for the implementation of the campaign. Ethical clearance was obtained from the institutional ethics committee of the *Fondation Congolaise pour la Recherche Médicale (FCRM)* for this study.

Preparatory work: work sessions were organized between the research team and the administrative representatives of the localities such as the sub-prefect, the leaders of villages and quarters. The leaders of villages and quarters were qualified as local research assistant. The aim of the work sessions was to better understand the epidemiological situation of the HAT in the districts, and to target the active foci of interest for the study. Careful planning and training of the research assistants was carried out to minimize on the cultural gap. Data collection: households were randomly selected and enrolment of volunteers was done after signing a written informed consent form. Focus group discussions (FGD) were composed of 10 participants per group. Thus, to each FGD, a structured questionnaire was administered including knowledge, attitude and practice questions with regard to HAT. In addition, participants were sensitized about the preventive measures and the benefits of the treatment against HAT including a presentation of WHO strategic plan for HAT eradication [7].

**Statistical analysis:** management and tabulation of raw data were carried out using Microsoft Excel (Microsoft Inc., Redmond, WA) version 2016. All statistical analyses were done using SPSS version 22.1. The normality of data distribution was checked using the Shapiro-Wilk test [8]. The absolute values and percentages were used to summarize descriptive statistics of the data. Chi-square test was used to assess relationships between selected and/or categorical variables. The level of significance was set at p<0.05.

**Ethical consideration:** this study received ethical clearance from the Institutional Ethics Committee of *Fondation Congolaise pour la Recherche Médicale* (Ethical Clearance N° 023/CIE/FCRM/2019), before starting the investigation. The confidentiality of the collected data was ensured. The volunteers signed an informed consent form prior to their enrolment in the study, and then the questionnaires were administrated.

## Results

**Study population:** a total of 220 adult volunteers were recruited and grouped into 22 FGDs in three districts including the district of Mpouya, Ngabe and Loudima (Figure 1(B,C,D)). More men (58.6%; 129/220) responded than women (41.4%; 91/220). All the women were engaged in farm activities, while men were primarily engaged in fishing, hunting and farming. Farm work (89.1%; 115/129) was the commonly undertaken activity, then fishing (66.7%; 86/129) and hunting (38.8%; 50/129). There was no significant difference in the number of FGDs (Chi^2^= 2.53; p=0.2824) that occurred in each district; Ngabe (45.4%; 10/22), Mpouya (36.4%; 8/22) and Loudima (18.2%; 4/22). As the individuals were grouped into focus group discussions, the district of Mpouya had one focus group discussion in each of the 8 selected villages. The district of Ngabe had one focus group discussion in each of the 7 selected villages and 3 FGDs in the one village. The district of Loudima only had 4 FGDs in the 4 selected villages.

**Perception of the population about the human Africa trypanosomiasis:** regarding the perception of HAT by FGDs, among four main answers, almost a half of the individuals of this study (48.2%) has described the HAT as a rural disease (Table 1), while 22.3% thought that witchcraft was the cause of the disease. Eleven percent and 8.6% considered the HAT as a bad luck and curse respectively. Twenty individuals (9.1%) did not know about HAT. The distribution of the answers about the perception of the HAT was different across the whole population, but dependent on the district (Khi^2^= 3.19, p = 0.203). When stratified by study site, the perception of the cause of HAT showed that the three main answers were rural disease (34.1%, 44.6 and 33.2%), curse (25.1%, 23.8% and 17.8%) and witchcraft (22.1%, 20.7% and 36.5%), given by the individuals of Mpouya, Ngabe and Loudima respectively. In all the districts, participants cited the main cause/definition of the disease as “rural” followed by “witchcraft”.

**Table 1:** perception about the human African trypanosomiasis in the study population

	Districts			Total (n=220)
Perceptions	Mpouya (n=80)	Ngabe (n=100)	Loudima (n=40)	
Rural disease % (n)	34.1 (27)	44.6 (44)	33.2 (13)	48.2 (106)
Curse % (n)	25.1 (20)	23.8 (24)	17.8 (7)	8.6 (19)
Witchcraft % (n)	22.1 (18)	20.7 (21)	36.5 (15)	22.3 (49)
Bad luck % (n)	11.4 (9)	8.2 (8)	6.6 (3)	11.8 (26)
Do not know % (n)	7.3 (6)	2.7 (3)	5.9 (2)	9.1(20)
Chi^2^				3.19
p-value				0.203

The Chi^2^ test was calculated to compare the distribution of answers by district; n: number of participants

**Factors affecting low participation in HAT diagnosis and knowledge about the clinical signs of the disease:** in this work, to the question “why people of your village are unwillingness to the screening of HAT?”, almost half of the individuals of this study (45.9%) responded that they were afraid of the lumbar puncture which is used during the sample collection. The fear of stigmatisation was reported by 22.3% (49/220) of the community. Sexual impotence and sick leave due to the pain caused by the puncture were both cited as reasons by 14% of respondents for not accepting screening (Table 2). Nine individuals (4.2%) gave other reasons like “it is a personal reason; they don´t want to answer etc.”. The distribution of the answers regarding reasons for the low attendance of HAT diagnostic screening was different in the whole population (Table 2), but no association was found with the district (Chi^2^= 2.252, p = 0.324).

**Table 2:** distribution of the reason of low participation to the human African trypanosomiasis diagnostic

	Districts			Total (n=220)
Reasons	Mpouya (n=80)	Ngabe (n=100)	Loudima (n=40)	
Fear of lumbar puncture % (n)	56.3 (45)	61.9 (62)	57.2 (23)	45.9 (101)
Stigmatisation % (n)	9.1 (7)	7.5 (8)	10.1 (4)	22.3 (49)
Sexual impotence % (n)	7.3 (6)	6.1 (6)	5.2 (2)	14 (31)
Sick leave % (n)	23.8 (19)	19.3 (19)	23.4 (9)	13.6 (30)
Others % (n)	3.5 (3)	5.2 (5)	4.1 (2)	4.2 (9)
Chi^2^				2.252
p-value				0.324

The Chi^2^ test was calculated to compare the distribution of answers by district; n: number of participants

**Figure 2:**
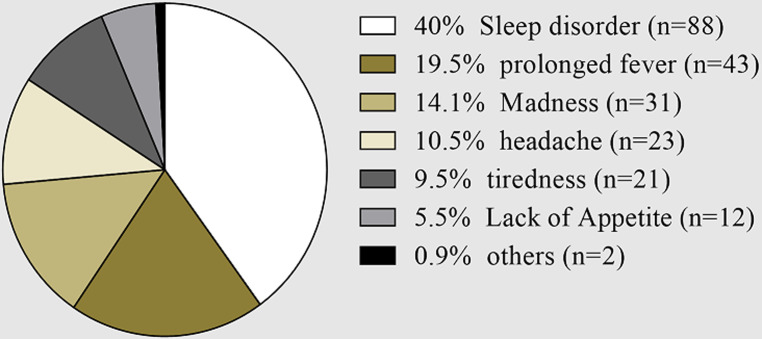
distribution of the respondents with respect of their Knowledge one major clinical signs of human Africa trypanosomiasis, the data were expressed as proportion (n: number of participants)

The participants were also questioned about their knowledge on major clinical signs of HAT (Figure 2). From the six major clinical signs cited by the individuals, sleep disorder (40%) was the more recurrent answer, followed by prolonged fever (19.5%), madness (14.1%), headache (10.5%), tiredness (9.5%) and lack of appetite (5.5%).

## Discussion

Human African trypanosomiasis (HAT) is one of the world´s classical neglected diseases which is endemic and a major threat to public health in the Republic of Congo. The recent progress in HAT surveillance and control means it is now possible to better estimate the number and distribution of individuals at risk of contracting the disease in RoC. The goal of the present study was to assess the possible socio-cultural factors associated with the low attendance of the Congolese population to the HAT screening in the foci at risk of the RoC. The participants recruited in this study were from either Mpouya, Ngabe or Loudima. The districts of Mpouya and Ngabé have been classified among the very high-risk areas, and the Loudima district a high-risk area for HAT, with *Tb gambiense* being the endemic pathogen [2,5].

Most participants were aware that HAT is a rural disease, and is found only in villages and not in larger urban settlements. This important point suggests that the relationship between environment and disease has been perceived. The second most reported perception was that HAT is a result of witchcraft or bad luck. This also could be explained by the fact that local culture, where there is widespread belief in spirits and health care is managed by traditional healers, is still commonly observed by the local population [9]. These results show that much is still unknown about HAT by the population in these endemic areas, and the misperception of the disease needs to be properly addressed. Other studies reported that the misperception of HAT was linked to the low level of education of respondents, likely a result of limited reading and comprehension of written information education and communication (IEC) materials [9,10]. Thus, the level of education needs to be taken into account when designing health interventions.

In addition to the fact that HAT is a neglected tropical infection, surveillance is challenged by the low response of the population to screening campaigns. In this study, the main reasons for low or non-participation of individuals to the diagnostic screening of HAT have been identified. It appears that the main problem is the diagnostic tool used for the screening itself; the lumbar puncture is an invasive and painful diagnostic tool. There is, therefore, a need to explore the use of non-invasive tool. The field-adapted card agglutination test for trypanosomiasis (CATT) serological technique, used by mobile teams for HAT mass screening in T. b. gambiense-infected areas can identify exposed individuals from the whole blood of a fingertip puncture [11]. However, immunological screening does not constitute the absolute proof of active infection as well as the stage of the disease. The determination of the disease stage (staging) is also crucial for the good management of the infected individuals. Staging still relies on a lumbar puncture which is painful and therefore unpopular among patients [12]. Sick leave as a result of HAT has an impact on family income and in a country where there is no health insurance; it is difficult for vulnerable populations to stop working.

The stigma of having HAT and the perceived sexual impotence it may cause were also cited in this study as reasons for the low attendance to HAT diagnostic testing. In their study carried out in South Sudan, Bukachi *et al*. (2018) have reported the following statement from the participants “if you are tested positive, your male organ will die”. However, the proportion of individuals giving this affirmation was not determined as part of this study. It is well known that the aspect of the male organ dying relates to reduced libido, hence inability to be sexually active. Exploring existing myths is important because they can be barriers to effective diagnosis and treatment of HAT. Addressing such issues using community dialogues is then crucial during the sensitization of the population [9].

The knowledge about the major clinical signs of the HAT was investigated in this study. Six major clinical signs including sleep disorder were cited. This finding demonstrates that, although the respondents have a misperception about HAT, they are aware of at least one major clinical signs of this disease. It is important to notice that this question was opened for giving the opportunity to the respondent to provide his/her answer. However, the fact that only 40% of respondents cited the sleep disorder as the major clinical sign highlights the need to improve the communication including education of the population at risk, since this sign is well known amongst the community as the common clinical manifestation [13].

One of the strengths of this study is related to the work with extensive experience of the utilization of the research tools amongst local populations used by local research assistants. The survey provides useful information on knowledge and perceptions in relation to HAT that can guide future HAT control interventions. However, the sample size was small and constitutes a limitation for generalization of the broad range of community perceptions on HAT.

## Conclusion

The present study reports that the main reason for not accepting the screening for HAT is the fear of lumber puncture as well as the stigmatisation of the patient by the community. The findings of this survey have important implications because it shows evidence that specific educational material should be created for this population and diagnostic tools developers must improve existing tools to be more reliable and less invasive to increase acceptability.

**Funding:** this study was supported by the Federal Ministry of Germany of the Development and Economic Cooperation (BMZ - Nr 2015.69.227 & BMZ - Nr 2016.68.797) awarded to the MNT project consortium. AL is funded through the Fondation Merieux. This work received also support from CANTAM (EDCTP-CSA2020NoE-3100). The funders did not play a role in the design of the study, collection, analysis, and interpretation of data, as well as the writing of the manuscript.

### What is known about this topic

- Trypanosoma brucei gambiense accounts for 95% of reported cases of human African trypanosomiasis (HAT) and causes a chronic infection in West and Central Africa;
- HAT is confined in foci located in sub-Saharan Africa, mainly in remote rural areas;
- The Republic of Congo is considered as an endemic country for HAT.

### What this study adds

- In remoted areas of the Republic of Congo most of the participants believed that the source of HAT was witchcraft or bad luck;
- The main reason for non-adherence to HAT screening was the fear of lumbar puncture and the stigmatization of having the disease.
